# Large-scale prediction of microRNA-disease associations by combinatorial prioritization algorithm

**DOI:** 10.1038/srep43792

**Published:** 2017-03-20

**Authors:** Hua Yu, Xiaojun Chen, Lu Lu

**Affiliations:** 1 Key Lab of Agricultural Biotechnology of Ningxia, Agricultural Biotechnology Center, Ningxia Academy of Agriculture and Forestry Sciences, 590 Huanghe East Road, Jinfeng District, Yinchuan, Ningxia, 750002, China.; 2 State Key Laboratory of Plant Genomics, Institute of Genetic and Developmental Biology, Chinese Academy of Sciences, No. 1 West Beichen Road, Chaoyang District, Beijing, 100101, China; 3 Beijing Computing Center, Beijing Academy of Science and Technology, Building 3 BeiKe Industrial park, Fengxian road 7, Haidian District, Beijing, 100094, China

## Abstract

Identification of the associations between microRNA molecules and human diseases from large-scale heterogeneous biological data is an important step for understanding the pathogenesis of diseases in microRNA level. However, experimental verification of microRNA-disease associations is expensive and time-consuming. To overcome the drawbacks of conventional experimental methods, we presented a combinatorial prioritization algorithm to predict the microRNA-disease associations. Importantly, our method can be used to predict microRNAs (diseases) associated with the diseases (microRNAs) without the known associated microRNAs (diseases). The predictive performance of our proposed approach was evaluated and verified by the internal cross-validations and external independent validations based on standard association datasets. The results demonstrate that our proposed method achieves the impressive performance for predicting the microRNA-disease association with the Area Under receiver operation characteristic Curve (AUC), 86.93%, which is indeed outperform the previous prediction methods. Particularly, we observed that the ensemble-based method by integrating the predictions of multiple algorithms can give more reliable and robust prediction than the single algorithm, with the AUC score improved to 92.26%. We applied our combinatorial prioritization algorithm to lung neoplasms and breast neoplasms, and revealed their top 30 microRNA candidates, which are in consistent with the published literatures and databases.

In biomedical research, unraveling the genome elements associated with the human diseases is still an important step for understanding the pathological mechanisms of diseases. In recent decades, plentiful achievements powered by the advanced next generation genome sequencing technologies have been made towards this objective^1^,^2^,^3^. Meanwhile, a variety of computational approaches have been proposed to predict the disease-related protein-coding genes using large-scale heterogeneous biological datasets^4^,^5^,^6^,^7^,^8^,^9^. For example, Kohler *et al*. and Franke *et al*. separately established a functional linkage network (FLN) from heterogeneous data sources, and utilized the FLN for prioritizing the genes related with various human diseases^4^,^5^. The successful identification of the disease-associated genes provides testable hypotheses for further experimental research, and therefore can effectively promote the understanding of pathogenesis of diseases.

MicroRNA genes are a special class of non-coding genes producing short non-coding microRNA molecules of ~22 nucleotides, which exert their functions mainly by suppressing the expression of target mRNAs at the post-transcriptional level^10^. In the past few years, many thousands of microRNA molecules have been discovered in various eukaryotic organisms by the experimental and computational methods^11^,^12^. MicroRNA recognizes their mRNAs by binding to the 3′ UTR regions of targets with imperfect complementary base pairing and thus causing target mRNAs cleavage or translation inhibition^13^. Plentiful study evidences have been exhibited that multiple microRNA molecules simultaneously bind to the same mRNAs, one microRNAs can also exert impacts on multiple target mRNA molecules and thus involve in a complex post-transcriptional regulatory network which plays the important roles in various important biological processes^14^,^15^,^16^. Since microRNA molecules participate in many crucial biological processes; it has been attracted more and more attentions^17^,^18^,^19^,^20^,^21^, a great number of microRNA-related works have been accomplished^22^,^23^,^24^,^25^,^26^,^27^. Interestingly, it has also been observed that the microRNA-mediated post-transcription regulations are often highly conserved during the evolution^28^,^29^. Furthermore, the accumulating biological studies have been showed that dysregulation of microRNAs are often related to the initiation, development and progression of complex diseases; especially; they are frequently involved in the human cancers^30^,^31^,^32^,^33^,^34^. Therefore, the system-level searching for the authentic associations between microRNAs and human diseases is an important topic of molecular oncology and biomedical research.

Although various biological experiments have been become available nowadays, experimental validation of disease-related microRNAs still remains challenging and expensive^35^,^36^,^37^. *In silico* computational methods for predicting the microRNA-disease associations are an alternative method to help selecting most reliable candidates for experimental validation. Nowadays, abundant microRNAs-disease association data has been generated rapidly during the studies of biomedicine and molecular biology. Jiang *et al*. and Lu *et al*. independently developed two publically available databases of miR2Disease and Human MicroRNA Disease Database (HMDD) based on the experimentally validated associations from the literatures^38^,^39^. Subsequently, Wang *et al*. constructed a manually collected mammalian non-coding RNA(ncRNA)-disease relationships (MNDR) database^40^ to provide a repository for exploring the functional roles of the ncRNAs in mammals. In addition, another publicly available database of Differentially Expressed MicroRNAs in human Cancers (dbDEMC) has been also built based on statistical analysis, which aim to provide the potential cancer-related microRNAs^41^. These datasets provided a solid basis for developing appropriate computational tools to uncover the complex links between microRNAs and diseases in a large scale.

Recently, Zou *et al*. reviewed and summarized the existing computational approaches for disease-related microRNAs prediction^42^,^43^. Among these, the machine-learning- based models and similarity-measure-based models are two main representatives. In the field of machine learning, several methods have been proposed to identify the disease-associated microRNAs. For example, the lasso regression model of protein- protein interaction were employed to discover the associations between microRNAs and diseases^44^. Jiang *et al*. designed a disease-associated microRNAs classifier by integrating the different genomic data source into a framework of Naïve Bayes model^45^. In addition to this model, Jiang *et al*. also used the classical Support Vector Machine (SVM) learning to distinguish the positive microRNA-disease associations from the negative ones^46^. Xu *et al*. proposed a machine learning method to prioritize the disease-related microRNAs by the topological features of the microRNA target- dysregulated network^47^. Zeng *et al*. employed two multi-path mathematical models to predict microRNA-disease associations using heterogeneous biological networks^48^,^49^. However, all these machine learning methods require the experimentally verified non-association information between the microRNAs and the diseases, which are quite difficult and even impossible to obtain. To overcome this existing limitation, Chen *et al*. proposed a semi-supervised learning method called Regularized Least Squares for microRNA-Disease Associations (RLSMDA) to uncover the associations between microRNAs and diseases^50^.

The similarity measure-based methods were proposed based on the observation that functionally related microRNA molecules tend to be associated with phenotypically similar diseases. For example, Zhang *et al*. first developed a statistical method to identify the cardiovascular disease associated microRNAs using the disease-related genes, Gene Ontology, microRNAs cluster and family analysis^51^. Li *et al*. predicted the microRNAs related to a specific disease by calculating the functional consistency score (FCS) among their targets and the known targets associated with the disease^52^. Jiang *et al*. proposed two computational methods by integrating different biological networks for prioritizing the disease related microRNAs and predicting the potential associations between microRNAs and diseases^53^,^54^. Chen *et al*. implemented the random walk algorithm on microRNA functional similarity network and developed the Random Walk with Restart for MicroRNA-Disease Association (RWRMDA) to predict the associations between microRNAs and diseases^55^. Xuan *et al*. developed the weighted K-most similar neighbors (HDMP) to infer the microRNAs associated with human diseases based on the microRNA functional similarity network derived from the disease semantic and phenotypic similarity, microRNA family and cluster data^56^. However, all these methods have the different inherent weaknesses. Firstly, some methods require microRNA targets and known genes related to disease which might be incomplete or inaccurate (such as the Jiang’s method). Secondly, the local similarity search strategy has adopted in several models, which only considered the node neighbor information for scoring the potential associations (such as the HDMP method). Thirdly, some prediction tools cannot be applied to discover the microRNAs related to the diseases without the known related microRNAs (such as the RWRMDA method). Considering the weaknesses in previous methods, Chen *et al*. proposed two global similarity search methods include the within and between score for predicting microRNA-disease association (WBSMDA) and the heterogeneous graph inference to recover the potential microRNA-disease associations (HGIMDA)^57^,^58^. Chen *et al*. also developed a mathematic model for predicting the different types of associations between microRNAs and diseases using the Restricted Boltzmann Machine^59^. Based on the standpoint of global network consistency, Chen *et al*. presented the network- consistency-based inference (NetCBI) for targeting the true microRNA-disease links^60^. Gu *et al*. further developed the network consistency projection for mining the links between microRNAs and diseases (NCPMDA). This method integrated microRNA similarity, microRNA family, disease similarity and microRNA-disease associations in a system level for scoring the probability of a microRNA and a disease was related^61^. In addition, some other tools have also been designed for uncovering the potential microRNA-disease associations^62^,^63^,^64^,^65^,^66^,^67^,^68^. While these proposed methods can effectively facilitate for future researches of the microRNAs involved in the pathogenesis of diseases, their performance has not been quite perfect.

In this study, we designed a combinatorial prioritization algorithm to infer the novel associations between microRNAs and diseases by modifying the existing maximizing information flow method, which was primarily designed for the disease-associated protein-coding gene prioritization. Importantly, this method can be applied to infer the novel microRNAs (diseases) for diseases (microRNAs) without the known related microRNAs (diseases). Validation experiments and case studies demonstrate that our method has the superior ability for predicting the novel links between microRNAs and diseases. Particularly, we observe that an ensemble-based predictor by combining the predictions of multiple inference methods can enhance the performance to a great extent. Our methods can uncover the accurate microRNA-disease associations and thus provide useful resources for identifying the novel links between microRNAs and diseases.

## Results

### Performance evaluation

To effectively evaluate the capability of combinatorial prioritization algorithm for inferring the potential microRNA-disease associations, we performed the parameter optimization process by grid search using leave-one-out cross-validation carried out on the known experimentally verified microRNA-disease associations (see Materials and Methods). In this process, all the remaining associations between microRNAs and diseases after removing the query microRNA (disease) associated disease (microRNA) were used as a training set to construct the network flow model. In the grid search process, we set the *α* from 0.1 to 0.7 with step 0.1, *β* from 0.1 to 0.7 with step 0.1, *γ* equals 1, 10, 100, 1000 and 10000 respectively, *η* from 1 to 10 with step 1, *σ* from 1 to 10 with step 1. After a comprehensive searching, the parameters (*α* = 0.1, *β* = 0.6, *γ* = 100, *η* = 6, *σ* = 10 for prioritizing the microRNAs related to the specific diseases in the leave-one-out cross-validation; *α* = 0.5, *β* = 0.1, *γ* = 1, *η* = 1, *σ* = 10 for prioritizing the microRNAs related to the specific diseases in the *ab initio* cross-validation; *α* = 0.5, *β* = 0.7, *γ* = 1, *η* = 1, *σ* = 4 for prioritizing the diseases related to the specific microRNAs in the leave-one-out cross-validation and *α* = 0.6, *β* = 0.3, *γ* = 1, *η* = 1, *σ* = 4 for prioritizing the diseases related to the specific microRNAs in the *ab initio* cross-validation) lead to best Area Under Curve (AUC) score of Receiver Operation Characteristic (ROC) curve were selected for further performance evaluation and comparison.

**Figure 1:**
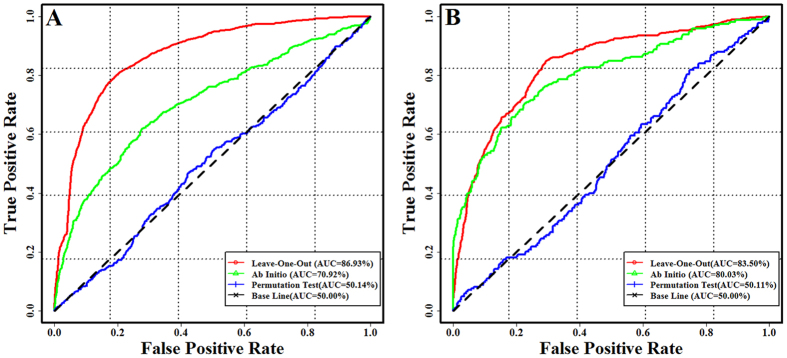
Performance of our combinatorial prioritization algorithm. (**A**) The ROC curves for prioritizing the microRNAs related to the specific diseases. (**B**) The ROC curves for prioritizing the diseases related to the specific microRNAs.

After obtaining the optimized parameters, we performed a series of leave-one-out cross-validation experiments to evaluate the capacity of our proposed approach for prioritizing the microRNAs related to the specific diseases (microRNA prioritization). The results are presented in Fig. 1A and Table 1, in which we can observe that our method is highly effective in uncovering the known microRNAs associated with the given query diseases. For instance, in the validation experiment using 300 randomly selected microRNAs as candidate microRNAs, the AUC score is as high as 86.76%. Meanwhile, it can be also observed that the performances obtained using our model is quite robust to the number of randomly selected candidate microRNAs. It is possible that the multiple associations between a microRNA and all its related diseases facilitate the inferring of novel diseases that are related to the microRNA. To demonstrate that our flow model is applicable to the diseases without the known related microRNAs, we conducted a series of *ab initio* cross-validation experiments by deleting the all links between the query diseases and their related microRNAs. When our model was tested using 300 randomly selected microRNAs as the candidate microRNAs, the highest AUC score, 71.30%, was obtained (Table 1), suggesting that our model is a powerful tool to achieve the goal of predicting the microRNA-disease associations for the diseases without the any known associated microRNAs.

To assess the ability of our method for prioritizing the diseases related to the specific microRNAs (disease prioritization), we further carried out a series of leave-one-out cross-validations and *ab initio* cross-validations using the randomly selected diseases as candidate diseases. The results are showed in Fig. 1B and Table 1, from which we can see the effectiveness and robustness of our method in predicting diseases that are associated with a query microRNA. For example, in the validation experiment using 130 randomly selected diseases as candidate diseases, we obtain the AUC scores of 81.52% and 79.63% in leave-one-out cross-validation and *ab initio* cross-validation, respectively. These results above suggest that our proposed method can successfully place the diseases that are truly related to the query microRNA at the top of all the candidate diseases. We have also investigated the effects of microRNA functional similarity measurements on the model’s inference capability. We built a new network flow model using another microRNA functional similarity network, which is derived from the microRNA’s target gene similarities. Using 20 different ratios of all inferred microRNA-disease links as prediction results, the paired single tail *t*-test for the AUC score comparison was carried out. The result shows that the microRNA functional similarity derived from disease phenotypic and semantic similarity are better for improving the inference capability of our method than which derived from their target similarity (*p*-value = 2.61E-2 for prioritizing the microRNAs related to the specific diseases, *p*-value = 1.74E-2 for prioritizing the diseases related to the specific microRNAs).

After confirming the usefulness of our method using the internal leave-one-out and *ab initio* cross-validations, we adopted the external independent validation to further assess the generalization ability of the models. In this experiment, the flow threshold which achieves the highest F1 score of 0.78 in the leave-one-out cross-validation was employed to make the predictions. The two network flow models for microRNA and disease prioritizations were applied to predict the unknown associations between all possible microRNAs and diseases. The results demonstrate that among 2163 and 724 associations between microRNAs and diseases predicted by these two models, 370 and 65 associations are now annotated in at least one of three databases of HMDD, miR2Disease and dbDEMC. The hypergeometric test was employed to calculate the probability of the chanciness of the obtained predictions. The extremely significant *p*-values of 2.18E-23 and 4.93E-135 are obtained, respectively. These results further revealed that our model has the strong potential to infer the novel microRNA-disease associations.

We substituted an undirected link between microRNA and disease with two directed links in the opposite orientation to allow for information pass in both directions when constructing the microRNAome-phenome network. It is possible that all connections between the microRNAome and the phenome flowing from diseases (microRNAs) to microRNAs (diseases) but not in the opposite direction could effectively improve the inference ability of the models. To study this, we removed all links from microRNAs to diseases (all links from diseases to microRNAs) in the microRNAome-phenome network and repeated the leave-one-out cross-validation for microRNA prioritization (disease prioritization). The results show that the AUC scores are 88.82% and 81.85% in identifying the microRNAs related to the specific diseases and in identifying the diseases related to the specific microRNAs, respectively. Compared with the previous analysis, where the connections pointing from both diseases to microRNAs and microRNAs to diseases (AUC scores equal 86.93% and 83.50%, respectively), we can only observe the relative slight fluctuation in the performances. Based on this, we conclude that the predictive performance of our proposed method is robust to the building method of the microRNAome-phenome network.

To further verify the superior predictive performance of our method is indeed owing to the biological connectivity information of microRNAome-phenome network, we conducted the permutation experiment by the following three steps i) permuting the associations between diseases and microRNAs while making the whole number of links between diseases and microRNAs unchangeable, ii) permuting the similarities in microRNAome while making the number of links of the microRNAome network unchangeable and iii) permuting the similarities in the phenome while making the number of links of the phenome network unchangeable. We repeated leave-one-out cross-validations 100 times using the permutated networks. The AUC scores are all around 50% (Fig. 1). Additionally, we also examined our model considering only the single aspect permutation including 1) without the microRNA similarity information, 2) without the disease similarity information and 3) without the associations between microRNAs and diseases. In the first case, the AUC scores from 86.93% drop down to 80.31% and from 83.50% decrease to 78.26% for the microRNA prioritization and disease prioritization, respectively. In the second case, we observe that the AUC scores from 86.93% reduce to 82.57% and from 83.50% go down to 77.34% for the microRNA prioritization and disease prioritization, respectively. If we remove the links between microRNAs and diseases, the performance is dramatically reduced, with the AUC scores pulled down to 71.58% and 70.41% in microRNA prioritization and disease prioritization, respectively. All these results suggest that the authentic biology relationships contained in the microRNAome-phenome network contribute to the successful prediction of microRNA-disease associations.

### Effects of parameters

In the process of constructing the flow models, five parameters affected the inference ability of our proposed approach. The parameter *α* (Alpha, ranging from 0 to 1) was used to decide the minimum functional similarity between two microRNAs and controlled the information quantity that can send from one microRNA to one another. The parameter *β* (Beta, ranging from 0 to 1) decided the minimum semantic and phenotypic similarity between two diseases. Therefore, it can affect the information quantity that can send from one disease to one another. The parameters *γ* (Gamma), *η* (Eta) and *σ* (Sigma) changing from 0 to the positive infinity, determined the capacity of the link going from the phenome (microRNAome) to the microRNAome (phenome) and controlled the information flow quantity that can be imported from the phenome to the microRNAome or vice versa. It was obviously not possible to enumerate all possible combinations of these five parameters. To effectively optimize the selection of the five parameters for advancing the predictive performance of our model, we analyzed each parameter individually while making the other parameters unchangeable in the framework of leave-one-out cross-validation.

By analyzing the distribution of functional similarity between microRNAs (diseases), we observed that the density of the similarity values appeared an obvious deflection point, indicating that most of the similarity values tend to be small (Fig. S1). We thus concluded that the parameters *α* and *β* should not be set too low to filter out the noise contained in microRNAome-phenome network. Based on this, we carried out a grid search process on these two parameters (*α* and *β* from 0.1007 to 0.8 with step 0.0007). The results are presented in Fig. S2. When the value of parameter *α* is set in a certain range, the AUC scores remain relatively stable. However, the predictive performance displays a significant drop down while the value of *α* exceed a given range. This is because that the biologically important connectivity information is not captured by our model when the parameters *α* be set too high, while too low value of *α* lead to the increase of noises included in the microRNA functional similarity network. These outcomes suggest the relatively small affecting of parameter *β* on the predictive performance of our proposed method. This is closely related to the vast majority of biological connectivity information is absorbed by our model when the parameter *β* change from 0.1007 to 0.8, since the most similarity scores of disease similarity network are lower than 0.1. These outcomes reveal that accurate similarity information is very important for effective detecting the potential microRNA-disease associations.

To study the effects of parameters *γ, η* and *σ*, we performed a grid search on different values of *γ* from 0.002 to 1 with the step 0.002 and from 2 to 501 with the step 1, *η* from 1.009 to 10 with the step 0.009 and *σ* from 1.009 to 10 with the step 0.009. As shown in Fig. S2, the AUC scores have a small change when the value of *γ* be set in a certain range, but the AUC scores fluctuate sharply while the value of *γ* is too high or too low. This is owing to 1) the similarity information in the microRNAome (phenome) will not be able to flow into the phenome (microRNAome) effectively if the value of *γ* be set too low; 2) the microRNA families and clusters information is not taken into account when the value of *γ* be set too high. On the contrary, our proposed method is quite steady in terms of the parameters of *η* and *δ* (Fig. S2). For example, when different parameter values *η* and *σ* are adopted in microRNAs prioritization, the AUC scores only change within 0.34% and 0.38% intervals in leave-one-out cross-validation, respectively. Similarly, the AUC scores fluctuate within 0.93% and 1.27% interval in diseases prioritization, respectively. These observations indicate that the parameter *γ* has a relatively large effect on the performance of our proposed model, while parameters *η* and *σ* can be given in an extensive range without affecting the performance of our method in a large extent.

### Performance comparison

Recently, a large number of computational tools have been proposed for the potential microRNA-disease association prediction. According to the published literatures, six methods including Jiang’s method^53^, HDMP^56^, RWRMDA^55^, Net-CBI^60^, RLSMDA^50^ and NCPMDA^61^ have been proposed as classical or state-of-the-art computational models. Since it has been shown that the performance of Net-CBI is obvious worse than RWRMDA^50^,^60^, we thus did not compare it with our method. We employed the leave-one-out cross-validation experiments for evaluating their ability for prioritizing the microRNAs related to the specific diseases based on the common gold standard association dataset as adopted in our study, and compared their performance with our proposed method using both the ROC curve and the Precision-Recall (PR) curve. Especially, the differences of inference capability of these algorithms were further analyzed by paired *t*-test. The paired *t*-test was performed by comparing the numeric vector of AUC scores obtained on 20 different proportions ranked lists of all inferred associations between microRNAs and diseases.

**Figure 2:**
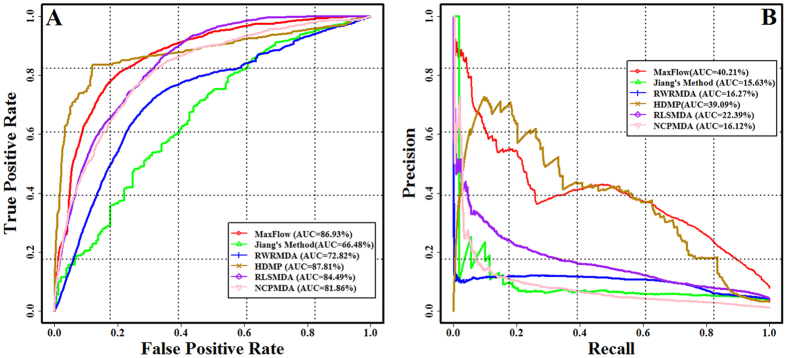
Comparison the performance of our method with the previous methods for prioritizing the microRNAs related to the specific diseases using the leave-one-out cross-validation experiments. (**A**) The ROC curve. (**B**) The PR curve.

Firstly, the ROC curve was employed to evaluate the performance of these *in silico* models. As shown in Fig. 2A, the ROC curve of our method (MaxFlow) lies clearly above those of Jiang’s method, RWRMDA, RLSMDA and NCPMDA. For Jiang’s method, the AUC score and the corresponding *p*-value are 66.48% and 3.61E-09, respectively. As shown in the manuscript of Jiang *et al*., this method uses the local similarity search and depend on the predicted targets of microRNAs, which results in a high false-positive and false-negative ratio. Other restrictions lie in the building of microRNA functional similarity network and using the simple Boolean phenotypical similarity between diseases (this method adopted only the information whether or not two microRNAs or diseases are similar, rather than their similarity scores). When the performance of our proposed method was compared with RWRMDA (the restart probability *r* of RWRMDA is set to 0.9 given by the experiments in the literature^55^), the AUC scores 72.82% for RWRMDA (*p*-value = 2.97E-07). This outcome is closely related to that this method predicts the microRNAs associated with a given disease by random walk only on the microRNA functional similarity network. The RLSMDA method (default parameters provided in the literature) achieves the AUC score of 84.49%, which is lower than our method (*p*-value = 3.82E-03). The NCPMDA method (default parameters provided in the literature) obtains an AUC score of 81.86%, which is also indeed smaller than our method (*p*-value = 2.84E-03). To compare with HDMP method, we set the number of most similar neighbors K = 20 which achieves the highest prediction performance in the author study. We did not find that the significant difference between our method and HDMP method (AUC score of ROC curve for HDMP = 87.81%, *p*-value = 0.36). However, the similarity network was not adopted in the HDMP method, which lead to it cannot be work for the isolate diseases without the known associated microRNAs. Our method can make prediction for the diseases without the known related microRNAs, which can overcome the key limitation of HDMP method.

Secondly, the performances of these methods were further assessed by the PR curve. It is clearly demonstrated that the PR curve of our method lies above those of Jiang’s method, RWRMDA, RLSMDA and NCPMDA in Fig. 2B. When comparing the AUC score of our method with Jiang’s method, RWRMDA, RLSMDA and NCPMDA, we can observe the apparent difference, with the *p*-values of 3.51E-10, 2.83E-8, 1.84E-5 and 3.61E-8. Similarly, we did not find that obvious difference between our method and HDMP (AUC score of PR curve for our method = 40.21%, AUC score of PR curve for HDMP = 39.09%, *p*-value = 0.43).

**Figure 3:**
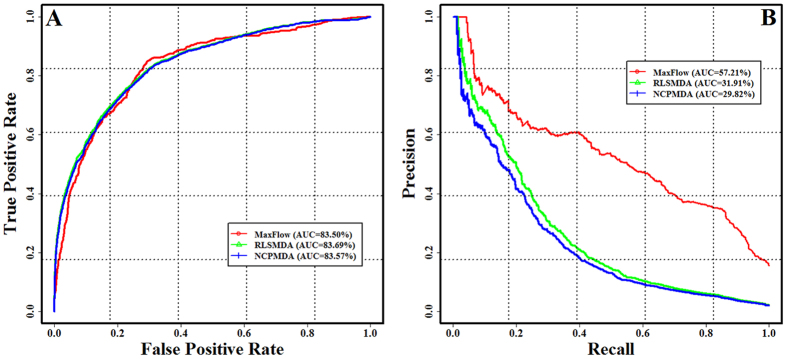
Comparison the performance of our method with RLSMDA and NCPMDA for prioritizing the diseases associated with the specific microRNAs using leave-one-out cross-validation experiments. (**A**) The ROC curve. (**B**) The PR curve.

In addition to comparing the performance of our method with the pervious methods for prioritizing the microRNAs related to the given diseases, we further carried out the leave-one-out cross-validation experiments to evaluate the predictive performance of these methods for prioritizing the diseases related to the specific microRNAs. We did not compare them with our method because that the Jiang’s method, HDMP and RWRMDA were designed for prioritizing the microRNAs associated with the specific diseases;. The ROC curves and PR curves of our method, RLSMDA and NCPMDA are presented in Fig. 3. For the ROC curves, we have not found the obvious difference among these methods, with the AUC score of 83.50%, 83.69% and 83.57% for our algorithm, RLSMDA and NCPMDA, respectively. The *p*-values are 0.82 and 0.69 in comparing our method with RLSMDA and NCPMDA, respectively. However, in terms of the PR curve, the predictive capability of our algorithm is indeed outperform RLSMDA and NCPMDA. The AUC scores are 57.21%, 31.91% and 29.82% for our algorithm, RLSMDA and NCPMDA, respectively. And the corresponding *p*-values are 3.57E-05 and 2.11E-05 in comparing our method with RLSMDA and NCPMDA, respectively.

RLSMDA and NCPMDA were implemented on the disease similarity network and microRNA similarity network simultaneously to predict the associations between microRNAs and diseases. These two methods used the similar data structure as our method. For the more fair and reasonable comparison, we implemented them on our miRNAome-phenome network for prioritizing the microRNAs related to the specific diseases and for prioritizing the diseases associated with the specific microRNAs. We performed the leave-one-out cross-validation experiments to evaluate the predictive performance of them. Interestingly, in the ROC curves (Fig. S3), we found that the AUC scores of RLSMDA and NCPMDA are improved to 86.21% and 86.70% for prioritizing the microRNAs related to the given diseases, and increased to 84.62% and 84.07% for prioritizing the diseases related to the specific microRNAs. While the AUC scores of these two methods have the certain rises, we did not observe that the significant differences between our method and them. When the performances were compared with the PR curve, we observed that the AUC scores of RLSMDA and NCPMDA are raised to 35.43% and 39.04% for prioritizing the microRNAs related to the given diseases, and ascended to 35.22% and 31.61% for prioritizing the diseases related to the specific microRNAs. However, the differences of predictive performance between our method and them are still apparent (see Fig. S3). These results suggest the network information flow model has the substantial contribution for improving the inference capability of a predictor.

We observed that the outcomes change largely from different inference models. The associations between microRNAs and diseases are correctly discerned in some models while indistinguishable in other methods. This indicated that each algorithm has its own strengths and weaknesses; no single algorithm can perform optimally across all datasets and performance measurements. Therefore, it is useful to build an ensemble predictor by aggregating the different computational methods. Here we adopted two ensemble methods, include weighted rank average and not weighted rank average^69^, to integrate the predictions of six algorithms mentioned above. The weight value of each method was given based on their AUC scores. In the ROC curves, the ensemble models get the AUC scores 92.26% and 91.21% for weighted rank average and not weighted rank average, which are outperformed the individual methods (Fig. S4). Moreover, we observe that the weighted rank average can obtain better performance than the not weighted rank average (*p*-value = 1.27E-2 for AUC scores of ROC curve, *p*-value = 2.46E-2 for AUC scores of PR curve). These results show that the ensemble-based method can improve the accuracy and robustness of a predictor.

### Case Studies

Previous studies have been shown that the microRNAs are located in neoplasms- associated genomic regions or brittle sites and are associated with the development of various cancers^70^,^71^,^72^. For instance, mir-215 and mir-301 are downregulated in colon cancer, and mir-129 is overexpressed in prostate cancer. Therefore, we adopted the combinatorial prioritization algorithm to identify both breast neoplasms and lung neoplasms related microRNAs. The obtained prediction results were validated by the newly reported microRNAs in HMDD database, miR2Disease database, dbDEMC database and published literatures. For simplicity, the top 30 microRNA candidates for these two cancers were selected here to illustrate our model’s application of predicting the potential disease-associated microRNAs (The detailed descriptions of the microRNA candidates of breast neoplasms and lung neoplasms were presented in the Table S1 and Table S2).

For breast neoplasms, there are 79 microRNA candidates which the rank ratio higher than the given threshold (0.78) derived from maximizing the F1-measure. The top 30 microRNA candidates and evidences for their association are illustrated in Table 2. 15 of them are identified to be related to breast neoplasms by the recently recorded microRNAs in HMDD database. 8 microRNAs are reported by the miR2disease database. The dbDEMC database includes 27 microRNAs which are up-regulated or down-regulated in breast cancer (malignant breast neoplasms). Two microRNAs are supported to have dysregulation in breast cancer by literatures^73^,^74^. Although we have not found any evidences in the databases and the literatures for hsa-mir-449b, 7 of top 50 predicted target genes of this microRNA are the breast cancer-associated genes, according to the G2SBC, a breast cancer database for genes to systems^75^. This indicates that hsa-mir-449b is likely to participate in the biological processes related to breast cancer. These results demonstrate that our method can discover the key breast neoplasms-related microRNAs.

82 microRNA candidates for lung neoplasms have the rank ratio larger than the given threshold of 0.78. Among the top 30 microRNA candidates, 11 microRNAs are sustained by the recently recorded microRNAs of HMDD database; 6 microRNAs are included in miR2Disease database; 23 microRNAs are contained in the dbDEMC database. Additionally, several published literatures also confirmed 3 microRNAs are significantly dysregulation in lung neoplasms^76^,^77^,^78^. Interestingly, we predicted a novel human lung cancer-related microRNA, has-mir-130a, which was reported in mouse and collected in the MNDR database^79^. For the remaining hsa-mir-208b, its target genes are related to the pathological processes of lung neoplasms^80^. These results well reveal that the top 30 microRNAs are the potential candidates associated with lung neoplasms.

Based on the results of case studies above, we performed the statistical analysis with the aim to further confirm the rationality and reliability of our method. Firstly, we selected and compared two groups of microRNA functional similarity datasets. The group one is comprised of the list of average similarity between each known cancer- related microRNA and all predicted cancer-related microRNAs. The group two is made up of the list of mean similarity between each known cancer-related microRNA and all other unrelated microRNAs. The extremely significant *p*-values of 6.22E-69 and 3.64E-12 are obtained for breast neoplasms and lung neoplasms by unpaired *t*-test, indicating that the microRNAs sharing more functional similarity with the known cancer-related microRNAs can be better predicted. Subsequently, we picked two groups of disease similarity datasets: I) The similarity score list between breast neoplasms (lung neoplasms) and the diseases that are associated with the predicted microRNAs of breast neoplasms (lung neoplasms); II) The similarity score list between breast neoplasms (lung neoplasms) and the diseases that are not related to the predicted microRNAs of breast neoplasms (lung neoplasms). Similarly, we got the extremely significant *p-*values of 2.85E-42 and 5.22E-7. This result is consistent with the principle of phenotypically similar diseases tend to be related to functionally related microRNAs. All above outcomes demonstrated that our method could be effective tools to directly explore the potential microRNA-disease associations.

## Discussion and Conclusions

In this article, we developed a combinatorial prioritization approach by integrating microRNA functional similarity network, disease semantic and phenotypic similarity network and known microRNA-disease associations into a phenome-microRNAome network to predict the novel microRNA-disease associations by maximizing network information flow. We demonstrated the effectiveness and robustness of our method for predicting the novel microRNA-disease associations by a suite of cross-validation experiments and independent validation experiment. We compared our method with 5 existing inference methods, which showed superior performance of our method over those existing methods. Case studies of breast neoplasms and lung neoplasms were implemented, and the top 30 microRNA candidates related to these two cancers are confirmed by databases and published literatures. Particularly, we showed that the ensemble-based approach by aggregating all these methods can remarkably upgrade the predictive performance. All these results demonstrated that our method provides the reliable microRNA-disease links for experimental validation, which can facilitate biomedical research of the microRNAs participating in the pathogenesis of diseases.

The success of our method full revealed that the strengths of associations between a query microRNA (disease) and the candidate diseases (microRNAs) can be largely captured by the maximum information flow transmitted from the query microRNA (disease) to the candidate diseases (microRNAs). Using the flow network model, our method integrated the different heterogeneous biological data sources to establish the microRNAome-phenome network and considered all paths between microRNAs and diseases. Such a system-level integration of the different types of biological datasets can effectively advance the model’s capability for inferring the associations between microRNAs and diseases. Firstly, microRNA functional similarity network was used to capture the biological relevance between two microRNAs. Secondly, the disease semantic and phenotypic similarity network was adopted to measure the pathological relevance between two diseases. Thirdly, the known microRNA-disease associations, microRNA cluster and family data were integrated to build the weighted network of microRNA-disease associations, which can accurately characterize biological links between microRNAs and diseases.

Although the maximizing information flow approach (MAXIF) has been adopted to prioritize the disease-associated protein-coding genes^7^, it has still not been employed to predict the microRNA-disease associations. Moreover, our method differs from the MAXIF method in the procedure of building heterogeneous biological network. The main innovations of our study are summarized as follows. I) Our method considers the global relevance between diseases and microRNAs, and thus can overcome the restrictions of the inference methods that rely on the local searching. II) Our method does not need the negative samples, which can effectively solve the problem that the current negative microRNA-disease associations are difficult or even impossible to obtain since there are no known non-associations between microRNAs and diseases. III) Our method can effectively predict the microRNAs (diseases) related to the given disease (microRNA) whose microRNAs (diseases) association information is not available. IV) We demonstrated that the ensemble-based method can obtain better predictions than those using single predictor. Although there are various advantages of our models, the limitations still exist. Our methods can be further improved in the following three aspects. I) The predictive capability of the network flow model can be enhanced by adding the updated high-quality microRNA-disease associations. II) Other bioinformatics data sources, such as the Gene Ontology (GO), gene expression profile and pathway information could be integrated to improve the model’s inference ability. In practice, this can be solved by incorporating multiple omics datasets into a unified network using the statistical technologies^81^,^82^,^83^. And III) more accurate and complete building of the microRNA functional similarity and disease semantic and phenotype similarity network could also effectively advance the performance of our proposed approach.

## Material and Methods

### Experimental dataset

The HMDD database created by Lu *et al*. includes 10237 associations among 572 microRNAs and 378 diseases collected from 3450 publications (June 20, 2013). The miR2Disease database contains 3273 associations between 349 microRNAs and 163 diseases (Mar 14, 2011). The updated MNDR database contains 202 Mus musculus microRNA-disease associations. The current version dbDEMC database is comprised of 590 mature microRNAs and 17 precursor microRNAs, which are differentially expressed in 14 cancers revealed by 48 biological experiments in the peer-reviewed publications. In this work, we downloaded the human microRNA-disease association dataset from the previous literature^56^, in which the associations were extracted from the HMDD database (November-2010 Version). In the meanwhile, the incorrect links with invalid names of the diseases and microRNAs were excluded. This dataset comprises 2076 associations among 338 microRNAs and 199 diseases, which were treated as the gold standard dataset for the performance evaluation of our proposed method in the validation experiments and the training dataset for inferring the novel microRNA-disease associations (see Dataset 1). Here we did not adopt the latest version of the data in HMDD and the datasets contained in other databases to build model because the microRNA-disease associations predicted by our models can be assessed by the novel associations introduced to HMDD and other databases. The microRNAs families and clusters information were obtained from the literature^39^, which contains 75 human microRNA families and 65 microRNA clusters (see Dataset 2).

The disease MeSH descriptors were extracted from the National Library of Medicine database (http://www.nlm.nih.gov/). There are 16 categories included in this database, for example, Category A for anatomic terms, Category B for organism terms and Category C for disease terms. We selected the relationships between diseases from the MeSH descriptors of Category C based on their Directed Acyclic Graph (DAG). Their phenotypic similarities were obtained by using the text mining analysis of the phenotype descriptions in Online Mendelian Inheritance in Man (OMIM) database^84^,^85^. Since the names of OMIM diseases are differently from those in MeSH, we extracted the mapping information from the comparative toxicogenomics database (http://ctdbase.org/) and searched against OMIM database for the OMIM number. The potential target genes of microRNA were predicted using the programs of PITA^86^, TargetScan^87^ and miSVR-miRanda^88^. In details, 4,095,752 associations among 677 microRNAs and 16,942 targets were retrieved from the PITA target catalog; 2,913,338 associations among 2318 microRNAs and 11,161 targets were obtained from the TargetScan. And 12,907,802 associations among 1100 microRNAs and 19885 targets were extracted from the www.microRNA.org, which was predicted by miSVR-miRanda.

### MicroRNA functional similarity network

Two microRNA functional similarity networks were used to assess the performance of our proposed method. On one hand, we obtained the known microRNA functional similarity network from the literature^56^. In this network, a similarity score for two microRNAs is calculated based on the hypothesis that microRNAs sharing similar biological roles are often associated with the phenotypically similar diseases. On other hand, we calculated the functional similarity between two microRNAs based on their target gene similarity. The functional similarity of every microRNA pair is defined as,


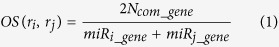


where *N*_*com_gene*_ is the number of shared targets of microRNA *i* and microRNA *j, miR*_*i_gene*_ and *miR*_*j_gene*_ is the number of targets of microRNA *i* and microRNA *j*, respectively. Based on the similarity score of each microRNA pair, the microRNA functional similarity network was constructed as a weighted graph, where vertexes represent the group of *n* microRNAs. Two vertexes *m*_*i*_ and *m*_*j*_ were connected by an edge in the network if they are functionally similar. Since most of low values of similarity scores are likely to be noise and only high scores have obvious biological meanings, we introduced a threshold *α* for this network, and only retaining the edges between microRNAs with the functional similarities that are larger than or equal to the threshold.

### Disease semantic and phenotypic similarity network

We inferred the disease semantic similarity based on the information content of each disease term^56^. Based the assumption that a more concrete disease contains the more information, the information content of disease MeSH term *S* can be defined as the negative log of the likelihood of it appearing in all DAGs of the diseases (*p(S)*), i.e. *IC(S) = *−log*(p(S))*. The semantic value of a disease *S* was then defined as,


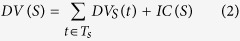


where *T*_*S*_ indicates the group of all ancestor diseases of *S*, including disease *S* itself. *DV*_*S*_*(t)* represents the semantic value of the parent disease *t* associated with disease *S*. Based on the hypothesis that the disease pairs sharing larger ratio of their DAGs are more conceptually similar, the semantic similarity between two diseases *A* and *B* can be then calculated as,


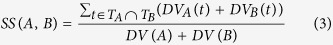


where *T*_*A*_ and *T*_*B*_ represent the groups of all ancestor diseases of *A* and *B*, including disease *A* and disease *B* themselves. *DV*_*A*_*(t)* and *DV*_*B*_*(t)* denote the semantic values of parent disease *t* associated with disease *A* and disease *B*, respectively.

Considering that phenotypic similarities do not contain all diseases, we incorporated the semantic similarity score and phenotypic similarity score between the diseases as their disease semantic and phenotypic similarity. For the disease pair with known OMIM number, the semantic and phenotypic similarity between them was defined as,


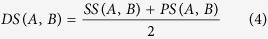


where *SS(A, B)* is the semantic similarity score between disease *A* and disease *B*, and *PS(A, B)* is the phenotypic similarity score between disease *A* and disease *B*. For the disease pair with unknown OMIM number, their semantic similarities were directly treated as semantic and phenotypic similarities. Based on this, we can obtain the disease semantic and phenotypic similarity network in which the vertexes denote diseases and the weighted edges indicate their similarities. To effectively filter the noise information of this network, we set a threshold *β* and only retaining the edges between diseases with the similarity values that are larger than or equal to the threshold.

### MicroRNA-disease association network

MicroRNA-disease association network was built by integrating microRNA-disease associations, microRNA family and cluster information. Firstly, we constructed a heterogeneous network consisting of two types of nodes based on the relationships between microRNAs and diseases. In the heterogeneous network, the nodes stand for either microRNAs or diseases, and links represent the associations between them. If a disease is known to be associated with a microRNA, we assigned a link between the microRNA and the disease. The weights of all edges in this heterogeneous network were set to *γ*. Subsequently, the members of microRNA families and clusters were assigned larger weights since they are more likely to associate with the similar diseases^89^. The allocation strategy is described as follows. To begin with, we defined the ratio of microRNAs associated with disease *d* for the *p*th family or cluster as,


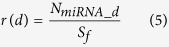


where *N*_*miRNA_d*_ is the number of disease *d*-related microRNAs of *p*th microRNA family or cluster, *S*_*f*_ is the size of *p*th microRNA family or cluster. In terms of the microRNAs pertaining to the *p*th family and a fraction of microRNAs in this family are related to disease *d*. The link weight of the microRNAs with respect to disease *d* in the *p*th family should be larger than *γ* and is calculated as,


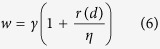


where *η* is a parameter for correcting the weight. With regard to the microRNAs in the microRNA clusters, we adopted the same weight assignment strategy as in the microRNA family. The link weight of the microRNAs in the *p*th cluster concerning *d* is defined as


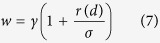


where *σ* is a factor for rectifying the weight. With respect to the microRNAs in both microRNA family and cluster, we assigned the link weight of the microRNAs with reference to *d* as,


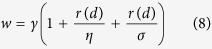


Based on the definitions above, we can get a weighted microRNA-disease association network, where vertex *M*_*i*_ and vertex *D*_*j*_ are linked if microRNA *i* is related to disease *j*.

### MicroRNAome-phenome network

MicroRNAome-phenome network was built by combining the microRNA functional similarity network, the disease semantic and phenotypic similarity network and the microRNA-disease association network. We refer to microRNA functional similarity network and disease semantic and phenotype similarity network as microRNAome and phenome, respectively. Obviously, the microRNAome-phenome network is a multi-layer network, whose nodes are the microRNAs or the diseases included in microRNAome and phenome, and whose links include the similarity relationships in microRNAome and phenome and the known associations between microRNAs and diseases. In this network, for each link (*u,v*), we defined a capacity function *f(u,v*) by assigning the weight value of each link as the capacity value *c(u,v*), which indicates the probability of two nodes are associated with each other. With these definitions, we denoted the microRNAome-phenome network as an undirected graph,


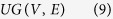


where *V* represents a group of either microRNAs or diseases and *E* is the group of weighted links indicating the capacity values flowing through microRNAs or diseases.

### Combinatorial prioritization algorithm

Network information flow model has been adopted in bioinformatics and systems biology^90^,^91^,^92^. Using this model, we designed a combinatorial prioritization algorithm to predict microRNA-disease associations. Given a query microRNA (disease), our goal is to prioritize diseases (microRNAs) that are related to this microRNA (disease) from a set of candidate diseases (microRNAs). To achieve this, for each combination of query disease (microRNA) and candidate microRNA (disease), we computed an association score by maximizing network information flow.

For microRNA prioritization, a given query disease *d*, the candidate microRNA set *S* and the microRNAome-phenome network *UG* were first imported into our model. The modeling procedure of our method was designed as follow.

(I) We substituted the undirected microRNAome-phenome network with a directed network by splitting each undirected links (*u, v*) into two distinct directed links (*u, v*) and (*u, v*).

(II) We modified and redefined the capacity formula between two vertexes as
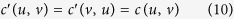


(III) Given a query disease *d*, we introduced a source node *s*, and assigned a directed link with an infinite capacity toward to it. Similarly, for every candidate microRNA contained in *S*, we introduced a sink node *t* and an infinite capacity and directed link pointing from this candidate microRNA to *t*. Thus, we got a new network *DG*.

(IV) In the new network, the capacity formula was redefined as
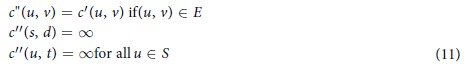


(V) The maximum information flow f^*^ from the source node *s* over all edges to the sink node *t* in the directed graph *DG* was calculated using the push-relabel algorithm^93^.

(VI) For each candidate microRNA *u* contained in *S*, we calculated the amount of flow leaving it as
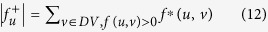
where *DV* represents the group of vertexes in the *DG*.

(VII) We used the flow quantity leaving microRNA *u* as a score to measure the extent of association between *u* and the query disease *d*. According to the obtained scores, the candidate microRNAs were sorted into to a ranked list.

**Figure 4:**
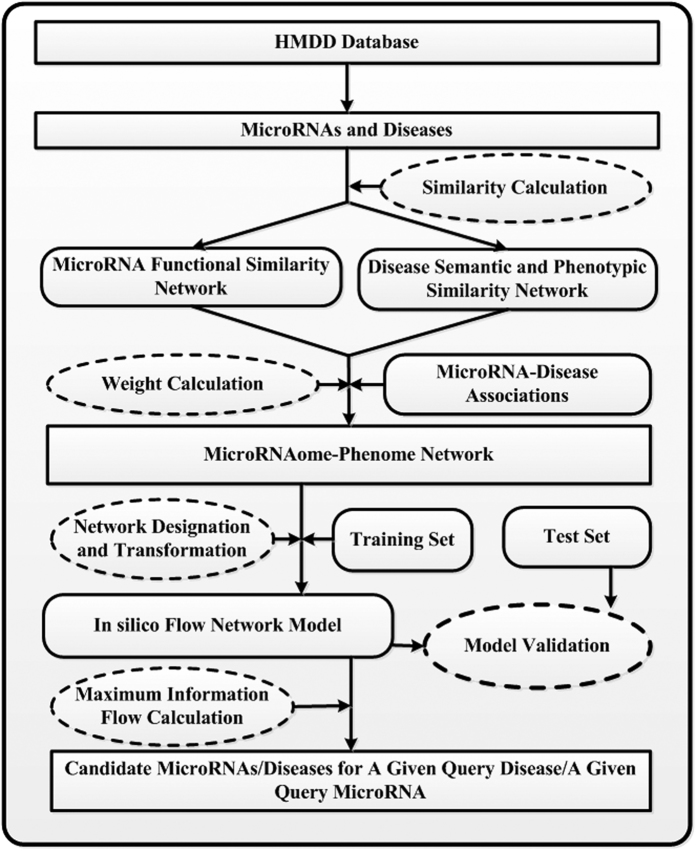
The flowchart of the whole modeling procedure.

For the disease prioritization, we reconstructed the information flow network model based on the microRNAome-phenome network by introducing a source node, a sink node, an infinite capacity and directed link pointing from the source node to the query microRNA and a series of infinite capacity and directed links pointing from the candidate diseases to the sink node. Based on this network structure and push-relabel maximum flow algorithm, we defined an association score for each candidate disease as the information flow leaving it and then ranked the diseases according to their scores. We multiplied all capacities in the flow network by a large number 1000 and round the resulting capacities to integers when using this algorithm. Figure 4 depicts the flowchart of the whole modeling procedure.

### Model validation and evaluation

To evaluate the performance of our proposed method in discovering microRNAs that were known to be related to the certain diseases from a set of candidate microRNAs, we implemented two types of cross-validation experiments. Experiment 1: Given a known association between a query disease and a microRNA in each run, a series of leave-one-out cross-validation experiments were conducted, in which we suppose the association was unknown and prioritized the microRNA against a series of candidate microRNAs. Experiment 2: we conducted a series of *ab initio* cross-validations to demonstrate that our network flow model is applicable to the isolate diseases without the known-associated microRNAs, in which we removed all known links between the query disease and their associated microRNAs, and prioritized the microRNA against a series of candidate microRNAs. Note the candidate microRNAs were randomly selected from the microRNA set, which was obtained from all microRNA in the benchmark dataset after deleting the query disease related microRNA (microRNAs). To assess the predictive performance of our approach in predicting diseases for the certain query microRNAs, we reconstructed the network flow model and performed the same cross-validation experiments (leave-one-out and *ab initio*) as adopted in the microRNA prioritization. Additionally, the external independent validations were also performed to evaluate the generalization ability of the obtained models. The newly reported microRNA-disease associations of the HMDD, miR2Disease and dbDEMC databases were used as the external independent validation dataset, in which we only selected the links whose microRNAs and diseases were included in the standard microRNA-disease association dataset for validation (November-2010 Version).

We adopted the Receiver Operation Characteristic (ROC) curve and Precision-Recall (PR) curve to evaluate the predictive performance of the proposed method. After each cross-validation experiment, we obtained a ranked microRNA-disease association list. We divided their ranks with the number of microRNA-disease pairs in the list to calculate rank ratios of microRNAs-disease pairs. Given a threshold of rank ratio, if the rank ratio of a microRNA-disease pair exceeds it, we considered the microRNA and the disease were associated. If an experimentally confirmed microRNA-disease association with rank ratio is higher than a given threshold, we considered it as successfully predicted. Based on these, we calculated the True Positive Ratio (TPR, also known as Sensitivity or Recall) as the proportion of successfully predicted microRNA-disease associations to all known microRNA-disease associations. The False Positive Ratio (FPR, also known as 1-specificity) was defined as the fraction of predicted negative microRNA-disease associations (i.e. the microRNA-disease pairs with the rank ratio higher than the given threshold but was not included in the known microRNA-disease associations) to all negative microRNA-disease associations. The Precision refer to the percentage of successfully predicted associations between microRNAs and diseases to all microRNA-disease associations with the rank ratio higher than the given threshold. We draw a Receiver Operating Characteristics (ROC) curve by changing the threshold and plotting the TPR (sensitivity) versus the FPR and then calculate the score of Area Under Curve. Similarly, the Precision-Recall (PR) curve was plotted by altering the threshold and plotting the Precision versus the Recall. Obviously, the larger AUC scores of ROC curve and PR curve indicate the higher performance of our proposed method. The F1 score defined below was adopted as performance indicator to obtain the optimal threshold


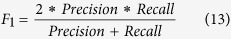


### Availability

The source codes of six inference methods including our combinatorial prioritization algorithm, Jiang’s method, RWRMDA, HDMP, RLSMDA and NCPMDA is freely available at https://github.com/huayu1111/M2DMiners.

## Additional Information

**How to cite this article**: Yu, H. *et al*. Large-scale prediction of microRNA-disease associations by combinatorial prioritization algorithm. *Sci. Rep.* **7**, 43792; doi: 10.1038/srep43792 (2017).

**Publisher's note:** Springer Nature remains neutral with regard to jurisdictional claims in published maps and institutional affiliations.

## Supplementary Material

Supplementary Information

Supplementary Dataset 1

Supplementary Dataset 2

## Figures and Tables

**Table 1:** The predictive performance of our method in a series of cross-validation experiments.

**Random MicroRNAs**	**Leave-One-Out**	50^a^	100^a^	150^a^	200^a^	250^a^	300^a^	All microRNAs^1^
		84.12%^c^	85.00%^c^	85.61%^c^	86.10%^c^	86.56%^c^	86.76%^c^	86.93%
	**Ab Initio**	50^a^	100^a^	150^a^	200^a^	250^a^	300^a^	All microRNAs^1^
		69.23%^c^	68.89%^c^	69.68%^c^	70.00%^c^	70.44%^c^	71.30%^c^	70.92%
**Random Diseases**	**Leave-One-Out**	90^b^	100^b^	110^b^	120^b^	130^b^	140^b^	All diseases^2^
		81.53%^c^	80.66%^c^	79.44%^c^	83.43%^c^	81.52%^c^	83.47%^c^	83.50%
	**Ab Initio**	90^b^	100^b^	110^b^	120^b^	130^b^	140^b^	All diseases^2^
		77.97%^c^	77.28%^c^	79.38%^c^	79.27%^c^	79.63%^c^	79.02%^c^	80.03%

^1^The candidate microRNAs were obtained after deleting the microRNA (microRNAs) related to query disease.

^2^The candidate diseases were obtained after deleting the disease (diseases) associated with the query microRNA.

^a^The number of randomly selected candidate microRNAs in microRNA prioritization.

^b^The number of randomly selected candidate diseases in disease prioritization.

^c^The AUC score of ROC curve.

**Table 2:** The top 30 breast neoplasms-related microRNA candidates.

Rank	MicroRNA Name	Evidences
1	hsa-let-7b	HMDD,dbDEMC
2	hsa-let-7c	HMDD,dbDEMC
3	hsa-mir-126	HMDD,dbDEMC,miR2Disease
4	hsa-mir-16	HMDD,dbDEMC
5	hsa-mir-100	HMDD,dbDEMC
6	hsa-let-7e	HMDD,dbDEMC
7	hsa-mir-135a	HMDD,dbDEMC
8	hsa-mir-130a	dbDEMC
9	hsa-let-7i	HMDD,dbDEMC,miR2Disease
10	hsa-mir-106a	dbDEMC
11	hsa-mir-150	dbDEMC
12	hsa-mir-181a	HMDD,dbDEMC,miR2Disease
13	hsa-mir-140	HMDD,dbDEMC
14	hsa-mir-203	HMDD,dbDEMC,miR2Disease
15	hsa-mir-192	dbDEMC
16	hsa-mir-138	dbDEMC
17	hsa-mir-191	HMDD,dbDEMC,miR2Disease
18	hsa-let-7g	HMDD,dbDEMC
19	hsa-mir-142	literature
20	hsa-mir-449a	literature
21	hsa-mir-101	dbDEMC,miR2Disease
22	hsa-mir-449b	G2SBC
23	hsa-mir-99b	dbDEMC
24	hsa-mir-186	dbDEMC
25	hsa-mir-372	dbDEMC
26	hsa-mir-95	dbDEMC
27	hsa-mir-371	dbDEMC
28	hsa-mir-152	HMDD,dbDEMC,miR2Disease
29	hsa-mir-148a	HMDD,dbDEMC,miR2Disease
30	hsa-mir-208	dbDEMC
