# Supplementary material for: Large-scale prediction of microRNA-disease associations by combinatorial prioritization algorithm
Source: Sci Rep. 2017 Mar 20;7:43792. doi: 10.1038/srep43792 (PMC5357838; doi:10.1038/srep43792)
Supplement: Supplementary Information [file srep43792-s1.pdf]

Supplementary Information

Large-scale prediction of microRNA-disease associations by combinatorial prioritization algorithm

Hua Yu[b], Lu Lu[c], Xiaojun Chen[a]

[a] Key Lab of Agricultural Biotechnology of Ningxia, Agricultural Biotechnology Center, Ningxia Academy of Agriculture and Forestry Sciences

[b] State Key Laboratory of Plant Genomics, Institute of Genetic and Developmental Biology, Chinese Academy of Sciences

[c] Beijing Computing Center, Beijing Academy of Science and Technology

[*] Corresponding author: Xiaojun Chen, smallgene@126.com

Supplementary file legends

Fig.S1 The distributions of similarity scores between microRNAs (diseases).

Fig.S2 Effects of five parameters for the performance of our proposed approach. (A) The distribution of AUC score for prioritizing the microRNAs related to the specific diseases. (B) The distribution of AUC score for prioritizing the diseases related to the specific microRNAs.

Fig.S3 Comparison the performance of our method with RLSMDA and NCPMDA for prioritizing the microRNAs related to the specific diseases and for prioritizing the diseases associated with the specific microRNAs using the same weighted networks. (A) The ROC curve for prioritizing the microRNAs related to the specific diseases. (B) The PR curve for prioritizing the microRNAs related to the specific diseases. (C) The ROC curve for prioritizing the diseases related to the specific microRNAs. (D) The PR curve for prioritizing the diseases related to the specific microRNAs.

Fig.S4 Effects of two different ensemble methods for the predictive performance. (A) Weighted rank average. (B) Not weighted rank average.

Table S1 The top 30 microRNA candidates for breast neoplasms in the ranked list.

Table S2 The top 30 microRNA candidates for lung neoplasms in the ranked list

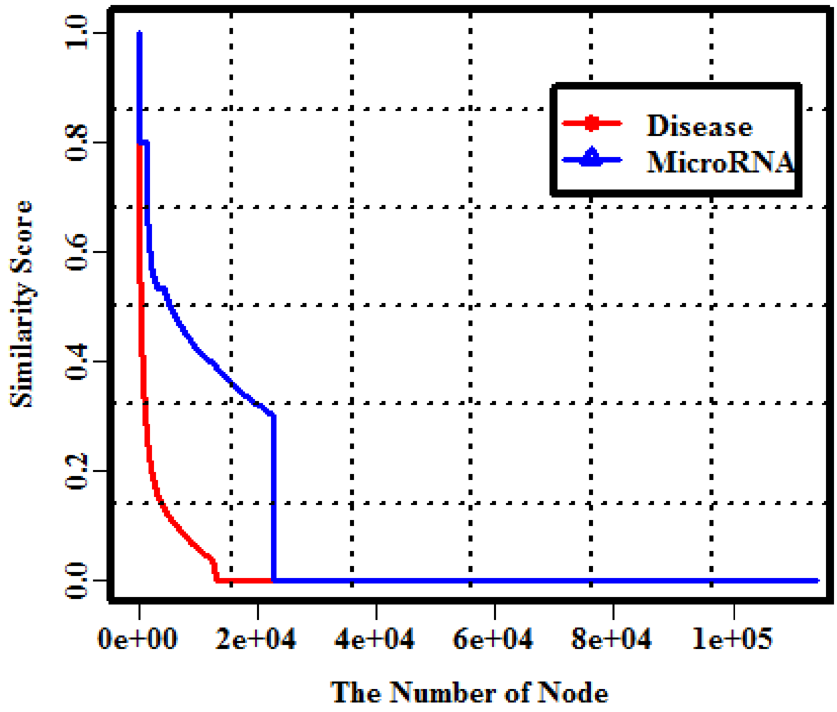

Fig. S1 The distributions of similarity scores between microRNAs (diseases).

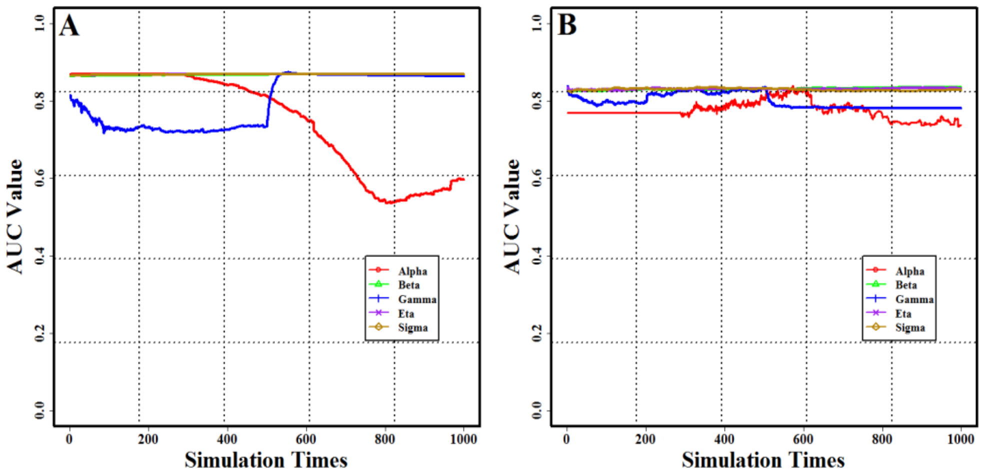

Fig.S2 Effects of five parameters for the performance of our proposed approach. (A) The distribution of AUC score for prioritizing the microRNAs related to the specific diseases. (B) The distribution of AUC score for prioritizing the diseases related to the specific microRNAs.

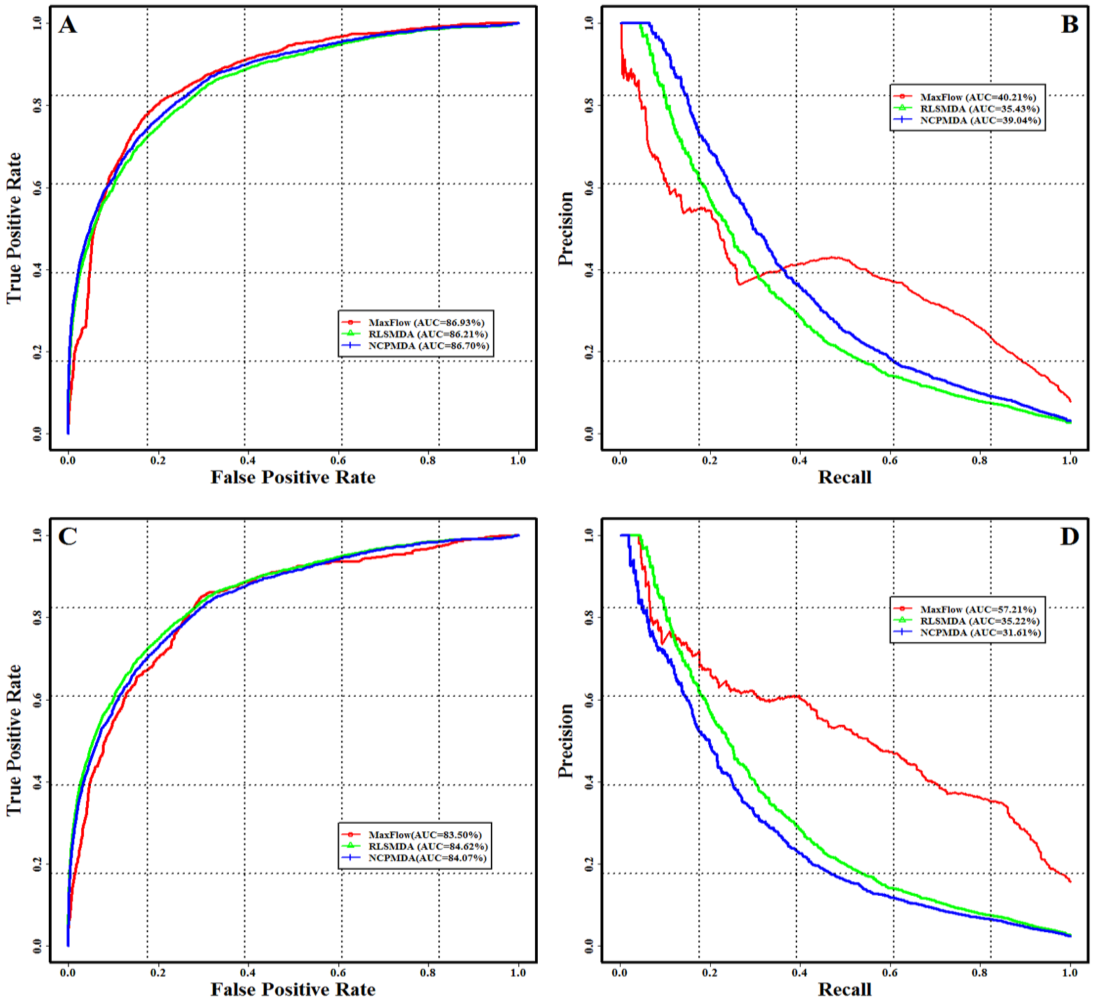

Fig.S3 Comparison the performance of our method with RLSMDA and NCPMDA for prioritizing the microRNAs related to the specific diseases and for prioritizing the diseases associated with the specific microRNAs implemented on the same weighted heterogeneous network. (A) The ROC curve for prioritizing the microRNAs related to the specific diseases. (B) The PR curve for prioritizing the microRNAs related to the specific diseases. (C) The ROC curve for prioritizing the diseases associated with the specific microRNAs. And (D) The PR curve for prioritizing the diseases associated with the specific microRNAs.

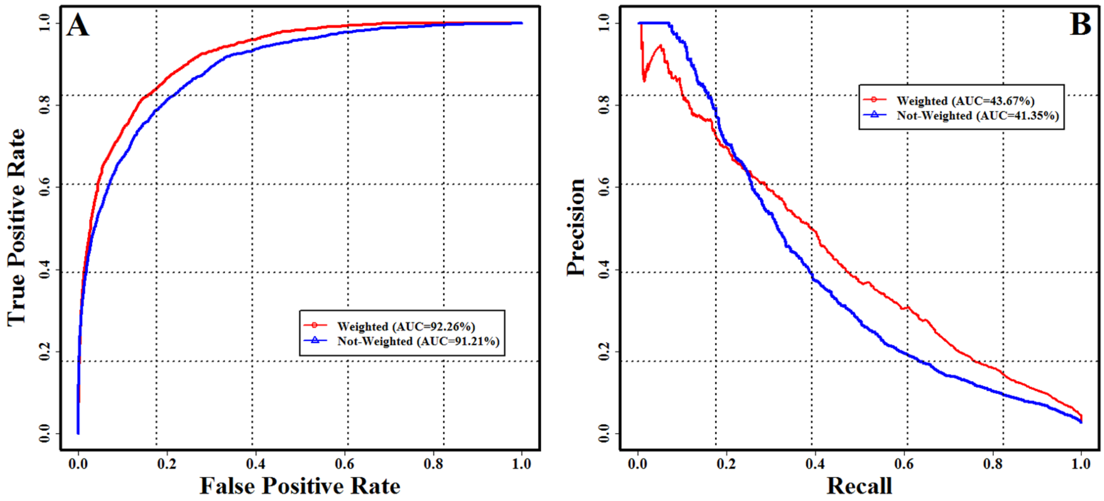

Fig.S4 Effects of two different ensemble methods for the predictive performance. (A) The ROC curve. (B) The PR curve.

Table S1 The top 30 microRNA candidates for breast neoplasms in the ranked list. (1) ‘literature’ means that there is a literature to support that the microRNA is related to human breast neoplasm. (2) With analysis of the microarray data sets, a microRNA is considered to have different express levels in breast cancer when compared to normal tissues. This kind of microRNAs is labeled by ‘dbDEMC’. (3) ‘HMDD’ means that a microRNA is a newly reported breast neoplasms-related microRNA which is collected by September-2012 version of human microRNA-disease database HMDD. (4) ‘miR2Disease’ means that a microRNA is included in miR2Disease database, a manually curated microRNA-disease association database. (5) G2SBC is a genes-to-systems breast cancer database, which is usually used for assistant studying the breast cancer. ‘G2SBC’ means some of the top predicted target mRNAs of a microRNA are breast cancer-related genes.

Rank	MicroRNA Name	Evidences	PMIDs	Descriptors
1	hsa-let-7b	HMDD<br>dbDEMC	22251626<br>18783629	Hsa-let-7b is a new reported breast neoplasms-related microRNA after the September-2012version of human-microRNA association database HMDD.<br>With the significance analysis of the microarrays, hsa-let-7b is identified as a potential microRNA down-regulated in breast cancer when compared to normal tissues
2	hsa-let-7c	HMDD<br>dbDEMC	21409395<br>17922911	Hsa-let-7c is a new reported breast neoplasms-related microRNA after the September-2012version of human-microRNA association database HMDD.<br>With the significance analysis of the microarrays, hsa-let-7c is identified as a potential microRNA down-regulated in breast cancer when compared to normal tissues.
3	hsa-mir-126	HMDD<br>dbDEMC<br>miR2Disease	21249429<br>16784538<br>18185580	Hsa-mir-126 is a new reported breast neoplasms-related microRNA after the September-2012version of human-microRNA association database HMDD.<br>With the significance analysis of the microarrays, hsa-mir-126 is identified as a potential microRNA down-regulated in breast cancer when compared to normal tissues.<br>Hsa-mir-126 is included in miR2Disease, a manually curated microRNA-disease association database. It means hsa-mir-126 is really associated with lung neoplasms.
4	hsa-mir-16	HMDD,<br>dbDEMC	22260523<br>17922911	Hsa-mir-16 is a new reported breast neoplasms-related microRNA after the September-2012version of human-microRNA association database HMDD.<br>With the significance analysis of the microarrays, hsa-mir-16 is identified as a potential microRNA up-regulated in breast cancer when compared to normal tissues.
5	hsa-mir-100	HMDD,<br>dbDEMC	21634028<br>17922911	Hsa-mir-100 is a new reported breast neoplasms-related microRNA after the September-2012version of human-microRNA association database HMDD.<br>With the significance analysis of the microarrays, hsa-mir-100 is identified as a potential microRNA down-regulated in breast cancer when compared to normal tissues.

6	hsa-let-7e	HMDD<br>dbDEMC	21969366<br>16784538	Has-let-7e is a new reported breast neoplasms-related microRNA after the September-2012version of human-microRNA association database HMDD.<br>With the significance analysis of the microarrays, hsa-let-7e is identified as a potential microRNA down-regulated in breast cancer when compared to normal tissues.
7	hsa-mir-135a	HMDD<br>dbDEMC	22439757<br>16784538	Has-mir-135a is a new reported breast neoplasms-related microRNA after the September-2012version of human-microRNA association database HMDD.<br>With the significance analysis of the microarrays, hsa-mir-135a is identified as a potential microRNA up-regulated in breast cancer when compared to normal tissues.
8	hsa-mir-130a	dbDEMC	15944708	With the significance analysis of the microarrays, hsa-mir-130a is identified as a potential microRNA down-regulated in breast cancer when compared to normal tissues.
9	hsa-let-7i	HMDD<br>dbDEMC<br>miR2Disease	22315424<br>16784538<br>16103053	Hsa-let-7i is a new reported breast neoplasms-related microRNA after the September-2012version of human-microRNA association database HMDD.<br>With the significance analysis of the microarrays, hsa-let-7i is identified as a potential microRNA down-regulated in breast cancer when compared to normal tissues.<br>Hsa-let-7i is included in miR2Disease, a manually curated microRNA-disease association database. It means has-let-7i is really associated with lung neoplasms.
10	hsa-mir-106a	dbDEMC	17922911	With the significance analysis of the microarrays, hsa-mir-106a is identified as a potential microRNA down-regulated in breast cancer when compared to normal tissues.
11	hsa-mir-150	dbDEMC	17922911	With the significance analysis of the microarrays, hsa-mir-150 is identified as a potential microRNA up-regulated in breast cancer when compared to normal tissues.
12	hsa-mir-181a	HMDD<br>dbDEMC<br>miR2Disease	23759567<br>17922911<br>19826037	Hsa-mir-181a is a new reported breast neoplasms-related microRNA after the September-2012version of human-microRNA association database HMDD.<br>With the significance analysis of the microarrays, hsa-mir-181a is identified as a potential microRNA up-regulated in breast cancer when compared to normal tissues.<br>Hsa-mir-181a is included in miR2Disease, a manually curated microRNA-disease association database. It means hsa-mir-181a is really associated with lung neoplasms.

13	hsa-mir-140	HMDD<br>dbDEMC	21953071<br>15944708	Has-mir-140 is a new reported breast neoplasms-related microRNA after the September-2012version of human-microRNA association database HMDD.<br>With the significance analysis of the microarrays, hsa-mir-140 is identified as a potential microRNA down-regulated in breast cancer when compared to normal tissues.
14	hsa-mir-203	HMDD<br>dbDEMC<br>miR2Disease	21553120<br>16784538<br>16103053	Hsa-mir-203 is a new reported breast neoplasms-related microRNA after the September-2012version of human-microRNA association database HMDD.<br>With the significance analysis of the microarrays, hsa-mir-203 is identified as a potential microRNA up-regulated in breast cancer when compared to normal tissues.<br>Hsa-mir-203 is included in miR2Disease, a manually curated microRNA-disease association database. It means hsa-mir-203 is really associated with lung neoplasms.
15	hsa-mir-192	dbDEMC	15944708	With the significance analysis of the microarrays, hsa-mir-181a is identified as a potential microRNA down-regulated in breast cancer when compared to normal tissues.
16	hsa-mir-138	dbDEMC	16784538	With the significance analysis of the microarrays, hsa-mir-181a is identified as a potential microRNA up-regulated in breast cancer when compared to normal tissues.
17	hsa-mir-191	HMDD<br>dbDEMC<br>miR2Disease	22898264<br>15944708<br>16103053	Hsa-mir-191 is a new reported breast neoplasms-related microRNA after the September-2012version of human-microRNA association database HMDD.<br>With the significance analysis of the microarrays, hsa-mir-191 is identified as a potential microRNA down-regulated in breast cancer when compared to normal tissues.<br>Hsa-mir-191 is included in miR2Disease, a manually curated microRNA-disease association database. It means hsa-mir-191 is really associated with lung neoplasms.
18	hsa-let-7g	HMDD<br>dbDEMC	21868760<br>15944708	Has-let-7g is a new reported breast neoplasms-related microRNA after the September-2012version of human-microRNA association database HMDD.<br>With the significance analysis of the microarrays, has-let-7g is identified as a potential microRNA down-regulated in breast cancer when compared to normal tissues.
19	hsa-mir-142	literature	16226311	Hsa-mir-142 is identified by microRNA expression profile as a potential microRNA related to breast cancer.
20	hsa-mir-449a	literature	19252524	Hsa-mir-449a is dys-regulated in breast cancer and thus suppresses breast cancer metastasis
21	hsa-mir-101	dbDEMC	15944708	With the significance analysis of the microarrays, hsa-mir-101 is identified

		miR2Disease	16103053	as a potential microRNA down-regulated in breast cancer when compared to normal tissues.<br>Hsa-mir-101 is included in miR2Disease, a manually curated microRNA-disease association database. It means hsa-mir-101 is really associated with lung neoplasms.
22	hsa-mir-449b	G2SBC	20525248	7 of top 50 predicted target genes of hsa-mir-449b are the breast cancer-related genes. This indicates that the microRNA is likely to participate in the breast cancer-related biological process.
23	hsa-mir-99b	dbDEMC	18783629	With the significance analysis of the microarrays, hsa-mir-99b is identified as a potential microRNA down-regulated in breast cancer when compared to normal tissues.
24	hsa-mir-186	dbDEMC	15944708	With the significance analysis of the microarrays, hsa-mir-186 is identified as a potential microRNA down-regulated in breast cancer when compared to normal tissues.
25	hsa-mir-372	dbDEMC	16784538	With the significance analysis of the microarrays, hsa-mir-372 is identified as a potential microRNA up-regulated in breast cancer when compared to normal tissues.
26	hsa-mir-95	dbDEMC	16784538	With the significance analysis of the microarrays, hsa-mir-95 is identified as a potential microRNA up-regulated in breast cancer when compared to normal tissues.
27	hsa-mir-371	dbDEMC	18783629	With the significance analysis of the microarrays, hsa-mir-371 is identified as a potential microRNA up-regulated in breast cancer when compared to normal tissues.
28	hsa-mir-152	HMDD<br>dbDEMC<br>miR2Disease	22935141<br>15944708<br>17948228	Hsa-mir-152 is a new reported breast neoplasms-related microRNA after the September-2012version of human-microRNA association database HMDD.<br>With the significance analysis of the microarrays, hsa-mir-152 is identified as a potential microRNA down-regulated in breast cancer when compared to normal tissues.<br>Hsa-mir-152 is included in miR2Disease, a manually curated microRNA-disease association database. It means hsa-mir-152 is really associated with lung neoplasms.
29	hsa-mir-148a	HMDD<br>dbDEMC<br>miR2Disease	22935141<br>17922911<br>17948228	Hsa-mir-148a is a new reported breast neoplasms-related microRNA after the September-2012version of human-microRNA association database HMDD.<br>With the significance analysis of the microarrays, hsa-mir-148a is identified as a potential microRNA up-regulated in breast cancer when compared to normal tissues.<br>Hsa-mir-148a is included in miR2Disease, a manually curated microRNA-disease association database. It means hsa-mir-148a is really associated with lung neoplasms.
30	hsa-mir-208	dbDEMC	16784538	With the significance analysis of the microarrays, hsa-mir-208 is identified as a potential microRNA up-regulated in breast cancer when compared to normal tissues.

Table S2 The top 30 microRNA candidates for lung neoplasms in the ranked list. (1) ‘literature’ means that there is a literature to support that the microRNA is related to human lung neoplasm. (2) With analysis of the microarray data sets, a microRNA is considered to have different express levels in lung cancer when compared to normal tissues. This kind of microRNAs is labeled by ‘dbDEMC’. (3) ‘HMDD’ means that a microRNA is a newly reported lung neoplasms-related microRNA which is collected by September-2012 version of human microRNA-disease database of HMDD. (4) ‘miR2Disease’ means that a microRNA is included in the miR2Disease, a manually curated microRNA-disease association database.

Rank	MicroRNA Name	Evidences	PMIDs	Descriptors
1	hsa-mir-106b	dbDEMC	19584273	With the significance analysis of the microarrays, hsa-mir-106b is identified as a potential microRNA up-regulated in lung cancer when compared to normal tissues.
2	hsa-mir-15a	dbDEMC	15944708	With the significance analysis of the microarrays, hsa-mir-15a is identified as a potential microRNA up-regulated in lung cancer when compared to normal tissues.
3	hsa-mir-133a	HMDD,<br>dbDEMC	22089643<br>17922911	Hsa-mir-133a is a new reported lung neoplasms-related microRNA after the November-2010 version of human-microRNA association database HMDD.<br>With the significance analysis of the microarrays, hsa-mir-133a is identified as a potential microRNA down-regulated in lung cancer when compared to normal tissues.
4	hsa-mir-10a	dbDEMC	15944708	With the significance analysis of the microarrays, hsa-mir-10a is identified as a potential microRNA down-regulated in lung cancer when compared to normal tissues.
5	hsa-mir-127	HMDD,<br>dbDEMC	22349807<br>15944708	Hsa-mir-127 is a new reported lung neoplasms-related microRNA after the November-2010 version of human-microRNA association database HMDD.<br>With the significance analysis of the microarrays, hsa-mir-127 is identified as a potential microRNA down-regulated in lung cancer when compared to normal tissues.
6	hsa-mir-100	HMDD,<br>dbDEMC	22120675<br>16784538	Hsa-mir-100 is a new reported lung neoplasms-related microRNA after the November-2010 version of human-microRNA association database HMDD.<br>With the significance analysis of the microarrays, hsa-mir-100 is identified as a potential microRNA down-regulated in lung cancer when compared to normal tissues.
7	hsa-mir-141	dbDEMC,<br>miR2Disease	15944708<br>19759262	With the significance analysis of the microarrays, hsa-mir-141 is identified as a potential microRNA down-regulated in lung cancer when compared to normal tissues.<br>Hsa-mir-141 is included in miR2Disease, a manually curated microRNA-disease association database. It means hsa-mir-141 is really associated with lung neoplasms.

8	hsa-mir-195	dbDEMC, miR2Disease	15944708 19654003	With the significance analysis of the microarrays, hsa-mir-195 is identified as a potential microRNA down-regulated in lung cancer when compared to normal tissues. Hsa-mir-195 is included in miR2Disease, a manually curated microRNA-disease association database. It means hsa-mir-195 is really associated with lung neoplasms.
9	hsa-mir-135a	HMDD, dbDEMC	23715500 19584273	Hsa-mir-135a is a new reported lung neoplasms-related microRNA after the November-2010 version of human-microRNA association database HMDD. With the significance analysis of the microarrays, hsa-mir-135a is identified as a potential microRNA down-regulated in lung cancer when compared to normal tissues.
10	hsa-mir-122	literature	21558792	Hsa-mir-122 is up-regulated in 5 of 8 different lung cancer patients after diagnosis.
11	hsa-mir-10b	HMDD, dbDEMC	22492962 15944708	Hsa-mir-10b is a new reported lung neoplasms-related microRNA after the November-2010 version of human-microRNA association database HMDD. With the significance analysis of the microarrays, hsa-mir-10b is identified as a potential microRNA down-regulated in lung cancer when compared to normal tissues.
12	hsa-mir-152	dbDEMC	15944708	With the significance analysis of the microarrays, hsa-mir-152 is identified as a potential microRNA down-regulated in lung cancer when compared to normal tissues.
13	hsa-mir-137	HMDD, dbDEMC	23178712 19584273	Hsa-mir-137 is a new reported lung neoplasms-related microRNA after the November-2010 version of human-microRNA association database HMDD. With the significance analysis of the microarrays, hsa-mir-137 is identified as a potential microRNA down-regulated in lung cancer when compared to normal tissues.
14	hsa-mir-16	dbDEMC, miR2Disease	15944708 19654003	With the significance analysis of the microarrays, hsa-mir-16 is identified as a potential microRNA down-regulated in lung cancer when compared to normal tissues. Hsa-mir-16 is included in miR2Disease, a manually curated microRNA-disease association database. It means hsa-mir-16 is really associated with lung neoplasms.
15	hsa-mir-130a	dbDEMC, miR2Disease	15944708 19654003	With the significance analysis of the microarrays, hsa-mir-130a is identified as a potential microRNA down-regulated in lung cancer when compared to normal tissues. Hsa-mir-130a is included in miR2Disease, a manually curated microRNA-disease association database. It means hsa-mir-130a is really associated with lung neoplasms.
16	hsa-mir-148b	dbDEMC	15944708	With the significance analysis of the microarrays, hsa-mir-148b is identified as a potential microRNA down-regulated in lung cancer when compared to normal tissues.

17	hsa-mir-151	literature	22303398	Hsa-mir-151 is up-regulated in the non-small cell lung carcinoma compared to non-tumorous tissue
18	hsa-mir-193b	dbDEMC	19584273	With the significance analysis of the microarrays, hsa-mir-193b is identified as a potential microRNA up-regulated in lung cancer when compared to normal tissues.
19	hsa-mir-27a	HMDD,<br>dbDEMC	23117485<br>19584273	Hsa-mir-27a is a new reported lung neoplasms-related microRNA after the November-2010 version of human-microRNA association database HMDD.<br>With the significance analysis of the microarrays, hsa-mir-27a is identified as a potential microRNA up-regulated in lung cancer when compared to normal tissues.
20	hsa-mir-206	HMDD	21157919	Hsa-mir-206 is a new reported lung neoplasms-related microRNA after the November-2010 version of human-microRNA association database HMDD.
21	hsa-mir-15b	dbDEMC	15944708	With the significance analysis of the microarrays, hsa-mir-15b is identified as a potential microRNA down-regulated in lung cancer when compared to normal tissues.
22	hsa-mir-153	dbDEMC	15944708	With the significance analysis of the microarrays, hsa-mir-153 is identified as a potential microRNA down-regulated in lung cancer when compared to normal tissues.
23	hsa-mir-208b	literature	20157481	Some target genes of hsa-mir-208b are the lung cancer-related genes. It shows that the microRNA is more likely to participate in the breast cancer-related biological process
24	hsa-mir-25	HMDD,<br>dbDEMC	22349819<br>19584273	Hsa-mir-25 is a new reported lung neoplasms-related microRNA after the November-2010 version of human-microRNA association database HMDD.<br>With the significance analysis of the microarrays, hsa-mir-25 is identified as a potential microRNA up-regulated in lung cancer when compared to normal tissues.
25	hsa-mir-139	dbDEMC,<br>miR2Disease	19584273<br>19654003	With the significance analysis of the microarrays, hsa-mir-139 is identified as a potential microRNA down-regulated in lung cancer when compared to normal tissues.<br>Hsa-mir-139 is included in miR2Disease, a manually curated microRNA-disease association database. It means hsa-mir-139 is really associated with lung neoplasms.
26	hsa-mir-584	literature	23006423	Genome-wide association study shown that hsa-mir-584 is potential related to lung cancer.
27	hsa-mir-1180	literature	20157481	Has-mir-1180's target gene CXCL12 is the lung cancer related gene. This indicates that the microRNA is likely to participate in the lung cancer-related biological process.
28	hsa-mir-1184	literature	20157481	Hsa-mir-1184's target gene PTPRT is the lung cancer related gene. This indicates that the microRNA is likely to participate in the lung cancer-related biological process.
29	hsa-mir-1246	literature	20157481	Hsa-mir-1246's target gene PTPRT is the lung cancer related gene. This

				indicates that the microRNA is likely to participate in the lung cancer-related biological process.
30	hsa-mir-1247	literature	20157481	Hsa-mir-1247's target genes IL12A and GHR are the lung cancer related genes. This indicates that the microRNA is likely to participate in the lung cancer-related biological process
